# Host Genetic Background Strongly Influences the Response to Influenza A Virus Infections

**DOI:** 10.1371/journal.pone.0004857

**Published:** 2009-03-18

**Authors:** Barkha Srivastava, Paulina Błażejewska, Manuela Heßmann, Dunja Bruder, Robert Geffers, Susanne Mauel, Achim D. Gruber, Klaus Schughart

**Affiliations:** 1 Department of Experimental Mouse Genetics, Helmholtz Centre for Infection Research & University of Veterinary Medicine Hannover, Braunschweig, Germany; 2 Research Group Immunoregulation, Helmholtz Centre for Infection Research, Braunschweig, Germany; 3 Gene Expression Analysis, Department of Cell Biology, Helmholtz Centre for Infection Research, Braunschweig, Germany; 4 Department of Veterinary Pathology, Faculty of Veterinary Medicine, Freie Universität Berlin, Berlin, Germany; University of Missouri-Kansas City, United States of America

## Abstract

The genetic make-up of the host has a major influence on its response to combat pathogens. For influenza A virus, several single gene mutations have been described which contribute to survival, the immune response and clearance of the pathogen by the host organism. Here, we have studied the influence of the genetic background to influenza A H1N1 (PR8) and H7N7 (SC35M) viruses. The seven inbred laboratory strains of mice analyzed exhibited different weight loss kinetics and survival rates after infection with PR8. Two strains in particular, DBA/2J and A/J, showed very high susceptibility to viral infections compared to all other strains. The LD_50_ to the influenza virus PR8 in DBA/2J mice was more than 1000-fold lower than in C57BL/6J mice. High susceptibility in DBA/2J mice was also observed after infection with influenza strain SC35M. In addition, infected DBA/2J mice showed a higher viral load in their lungs, elevated expression of cytokines and chemokines, and a more severe and extended lung pathology compared to infected C57BL/6J mice. These findings indicate a major contribution of the genetic background of the host to influenza A virus infections. The overall response in highly susceptible DBA/2J mice resembled the pathology described for infections with the highly virulent influenza H1N1-1918 and newly emerged H5N1 viruses.

## Introduction

Influenza A virus infections have caused multiple severe pandemics in recent human history. It is estimated that during the 1918 “Spanish flu” pandemics, about 50 million people died world-wide [1], and during the pandemics of 1957 and 1968, about 1 million people succumbed to influenza [2], [3]. Seasonal yearly epidemics are caused by variants of the subtypes H1N1 and H3N2 and kill about 1 million people per year world-wide [4]. Recently, a new subtype, H5N1, appeared which is highly pathogenic in birds and can be transmitted to humans that are in close contact with infected birds. H5N1 infections in humans cause a severe pneumonia that is fatal in about 50% of infected individuals [5], [6].

Intensive research has been performed on the virulence and evolution of the influenza virus [7], [8]. However, very little is known about the influence of specific genes or genetic backgrounds in humans that contribute to the susceptibility or resistance to influenza infections. The importance of host genetic factors in humans has been shown for several bacterial and viral pathogens [9]–[11]. An investigation of the influenza death records over the past 100 years in the population of Utah provided evidence for an increased risk in close and distant related relatives [12], although the analysis of influenza related deaths in the population of Iceland during the Spanish flu pandemic did not find any conclusive evidence for a genetic contribution [13]. It is important to note that it is very difficult to perform studies on host susceptibility to acute infections in humans due to the complexity of genetic variants and largely different environmental influences, such as nutrition, life style, medication, exposure to other pathogens, *etc*. Thus, a much better way to understand the principle mechanisms underlying susceptibility or resistance to infectious diseases is to use experimental animal model systems.

The mouse has been shown to represent a particularly useful model to study the virulence of the highly pathogenic H5N1 and the 1918 H1N1 influenza viruses [14], [15]. For less virulent virus subtypes, depletion of specific immune cell populations has demonstrated critical involvements of neutrophils [16], [17], macrophages [17], [18], dendritic cells [19], [20], natural killer cells [21], B cells [22], and T killer cells [23] during the host response.

Using mouse mutant strains, several mammalian genes have been shown to be important for the host defense against an influenza virus infection, that include the *Mx1*, *Stat1*, *Pkr*, *Ifnar1*, *and Ncr1* genes [21], [24]–[26]. However, it is obvious that this is only a very small fraction of the essential genes involved. A study performed by Crozat et al. in 2006 [27] estimates that about 480 genes are critical for the host defense against an infection with mouse cytomegalovirus, and more than 1000 genes changed their expression levels after infection with *Mycobacterium tuberculosis* [28] or influenza A virus [29].

From studies of genetic predisposition it has become clear that the host response is not only influenced by single genes but by combinations of genes and their variants. Thus, besides Mendelian (single) genes, also complex (multi-gene) genetic effects need to be analyzed to understand the full repertoire of host responses to pathogens. For mice, well defined genetic reference populations (GRP) exist that allow the analysis of complex genetic traits and the effect of multiple contributing gene loci. Mouse GRP are available as inbred laboratory and wild-derived mouse strains, recombinant inbred strains, interspecific recombinant inbred strains, chromosome substitution strains, and consomic strains [30]. These resources have been extensively used to identify quantitative traits and single gene loci contributing to the host response to infections with different pathogens (reviewed in *e.g.*, [31]–[37]). However, in most studies with influenza virus, only two strains of mice, BALB/c and C57BL/6, have been used, but have not been compared directly. To date only one study has made a direct comparison of the influence of genetic background on gene expression and susceptibility in BALB/cByJ versus C57BL/6J mice [29].

As a first step towards the analysis of complex genetic traits influencing resistance and susceptibility to influenza, we have investigated the host response to two virus subtypes in seven inbred laboratory mouse strains. Two mouse strains were identified which exhibited a very pronounced susceptibility to influenza infections. We then compared weight loss, survival, lung pathologies, cytokine/chemokine responses and virus replication between one of the highly susceptible strains, DBA/2J, and one of the more resistant strains, C57BL/6J. Whereas C57BL/6J mice could control virus replication and clear the infection, DBA/2J mice exhibited higher viral loads, higher levels of cytokines and chemokines, enhanced lung pathology, and were not able to clear the viral infection.

## Results

### Inbred mouse strains exhibit large differences in their response to influenza A virus

**Figure 1:**
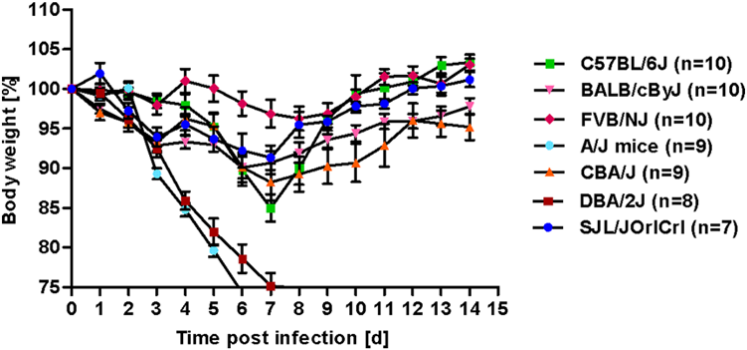
Different inbred laboratory mouse strains exhibit variable kinetics of weight loss and survival after infection with Influenza A virus. C57BL/6J, DBA/2J, FVB/NJ, CBA/J, BALB/cByJ, A/J and SJL/JOrlCrl mice were infected intra-nasally with 2×10^3^ FFU of PR8 virus. Weight loss and survival of infected mice was followed over a period of 14 days. Mortality also includes mice that were sacrificed because they had lost more than 25% of body weight. Mean percent of body weight change (±SEM) for each group of inbred strains is shown. For DBA/2J and C57BL/6J mice, data are from two independent experiments. Statistical analysis of pair wise comparisons for all strains and days are presented in table 1.

Seven different inbred laboratory mouse strains were infected with a dose of 2×10^3^ FFU of influenza A virus PR8 (H1N1) and followed for a period of 14 days after infection. As illustrated in figure 1, large differences in the kinetics of weight loss and survival were observed. Most notably, mice from two inbred strains, DBA/2J and A/J, lost weight very rapidly and died within the first seven days after infection, or were sacrificed because weight loss exceeded 25%. All infected mice from the other strains survived this infection dose. The weight loss in the highly susceptible strains DBA/2J and A/J mouse strains was significantly different compared to all resistant strains (table 1). The surviving strains exhibited three principle types of weight loss kinetics. BALB/cByJ, CBA/J and SJL/JOrlCrl rapidly lost weight within the first days after infection until about day 6 to 7 and then slowly recovered, with SJL/JOrlCrl being the least affected in this group (figure 1). C57BL/6J mice did not lose weight early after infection but rapidly lost weight after day 4 until day 7 and then quickly recovered (figure 1). The weight loss in C57BL/6J mice was significantly different to the BALB/c mouse strain on days 2–4 but not at later time points (table 1). FVB/NJ mice were the least affected by this infection dose (figure 1, table 1). The weight change in infected DBA/2J and C57BL/6J mice was significantly different compared to mock-infected mice, instilled with PBS only (data not shown).

**Table 1:** Pair wise statistical comparison of all mouse strains presented in figure 1.

DAY 2		C57BL/6J	DBA/2J	A/J	BALB/cByJ	FVB/NJ	CBA/J	SJL/JOrlCrl
	C57BL/6J		*	n.s	***	n.s	*	n.s
	DBA/2J			*	n.s	n.s	n.s	n.s
	A/J				***	n.s	*	*
	BALB/cByJ					**	n.s	n.s
	FVB/NJ						*	n.s
	CBA/J							n.s
	SJL/JOrlCrl							
DAY 3	C57BL/6J		***	***	***	n.s	**	**
	DBA/2J			*	n.s	**	n.s	n.s
	A/J				**	***	*	**
	BALB/cByJ					**	n.s	n.s
	FVB/NJ						*	*
	CBA/J							n.s
	SJL/JOrlCrl							
DAY 4	C57BL/6J		***	***	*	n.s	n.s	n.s
	DBA/2J			n.s	***	***	***	***
	A/J				***	***	***	***
	BALB/cByJ					***	n.s	n.s
	FVB/NJ						n.s	*
	CBA/J							n.s
	SJL/JOrlCrl							
DAY 5	C57BL/6J		***	***	n.s.	n.s	n.s	n.s
	DBA/2J			n.s	***	***	***	***
	A/J				***	***	***	***
	BALB/cByJ					**	n.s	n.s
	FVB/NJ						n.s	**
	CBA/J							n.s
	SJL/JOrlCrl							
DAY 6	C57BL/6J		**	***	n.s.	**	n.s	n.s
	DBA/2J			n.s	***	***	***	***
	A/J				***	***	***	***
	BALB/cByJ					**	n.s	n.s
	FVB/NJ						*	*
	CBA/J							n.s
	SJL/JOrlCrl							
DAY 7	C57BL/6J		***	***	*	***	n.s	n.s
	DBA/2J			n.d	***	***	***	***
	A/J				***	***	***	**
	BALB/cByJ					*	n.s	n.s
	FVB/NJ						*	*
	CBA/J							n.s
	SJL/JOrlCrl							
DAY 10	C57BL/6J				*	n.s	**	n.s
	BALB/cByJ					**	n.s	*
	FVB/NJ						***	n.s
	CBA/J							**
	SJL/JOrlCrl							
DAY 14	C57BL/6J				**	n.s	**	n.s
	BALB/cByJ					**	n.s	*
	FVB/NJ						**	n.s
	CBA/J							**
	SJL/JOrlCrl							

p-values for significance were calculated for all pair wise comparisons between strains and for all days shown in figure 1 using non-parametric Mann-Whitney-U-test. Day 3: the susceptible strain DBA/2J showed significant differences in weight loss compared to the resistant strains C57BL/6J (p<0.001) and FVB (p<0.01); the susceptible strain A/J showed significant weight loss compared to all resistant strains (BALB/c: p<0.01; FVB: p<0.001; CBA: p<0.05; SJL: p<0.01). Days 4, 5, 6, 7: the susceptible strains DBA/2J and A/J showed significant differences in weight loss compared to all resistant strains (in almost all cases with p<0,001; except for day 6 between DBA/2J and C57BL/6J: p<0.01). Strain C57BL/6J exhibited significant differences in weight loss compared to the BALB/c mouse strain at days 2–4 (p<0.001 at days 2 and 3, p<0.05 at day 4) but not at later time points. On days 6 and 7, FVB differed significantly from all other strains (p-values from <0.05 to <0.001).

* p value<0.05.

** p<0.01.

*** p value<0.001.

### LD_50_ and course of infection are very different between C57BL/6J and DBA/2J mouse strains

**Figure 2:**
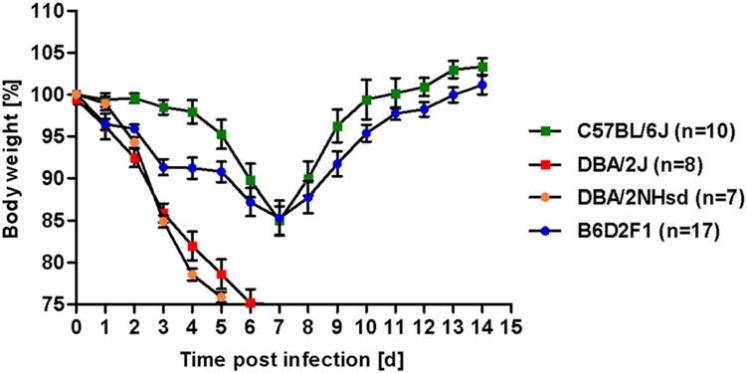
(C57BL/6J×DBA/2J) F1 mice display an intermediate phenotype after infection with Influenza A virus and DBA/2NHsd exhibit the same susceptibility as DBA/2J. C57BL/6J, DBA/2J, DBA/2NHsd and (C57BL/6J×DBA/2J) F1 (labeled B6D2F1 in the figure) mice were infected intra-nasally with 2×10^3^ FFU PR8 virus. Weight loss and survival of infected mice was followed over a period of 14 days. Mortality also includes mice that were sacrificed because they had lost more than 25% of body weight. Mean percent of body weight change (±SEM) are shown. For DBA/2J, C57BL/6J, and B6D2F1, data from two independent experiments were combined. p-values for significance were calculated for pair wise comparisons between all strains and for all days using non-parametric Mann-Whitney-U-test. The (C57BL/6J×DBA/2J) F1 group differed significantly in weight loss from days 2–4 when compared to the C57BL/6J group (p<0.001 on days 2, 3; p<0.01 on day 4) and from days 3–5 when compared to the DBA/2J and DBA/2NHsd groups (p<0.01). On days 2–5, DBA/2J and DBA/2NHsd strains differed significantly in their weight loss from C57BL/6J (p<0.001 for all cases, except p<0.01 at day 5 for DBA/2NHsd vs. C57BL/6J). Weight loss was not significantly different between the sub-strains DBA/2J and DBA/2NHsd groups for all days.

Hybrid mice, (C57BL/6J×DBA/2J) F1, showed an intermediate phenotype with a rapid weight loss within the first days after infection (figure 2). However, this weight loss was not as dramatic as for DBA/2J. It reached its peak on day 7, similar to the kinetics observed for C57BL/6J mice. After day 7, F1 hybrid mice recovered and weight gain was slightly delayed, compared to C57BL/6J mice.

The DBA/2J sub-strain is deficient in a specific subset of natural killer cells [38]. Therefore, another sub-strain of DBA, DBA/2NHsd, was studied which is able to generate this subset of NK cells. As shown in figure 2, the DBA/2NHsd sub-strain was as susceptible as DBA/2J. Thus, the high susceptibility in DBA mice is not due to this unique feature of the DBA/2J sub-strain.

To further investigate the host response between susceptible and resistant mouse strains, we selected one of the highly susceptible strains, DBA/2J, and one of the resistant strains, C57BL/6J for study in more detail.

**Figure 3:**
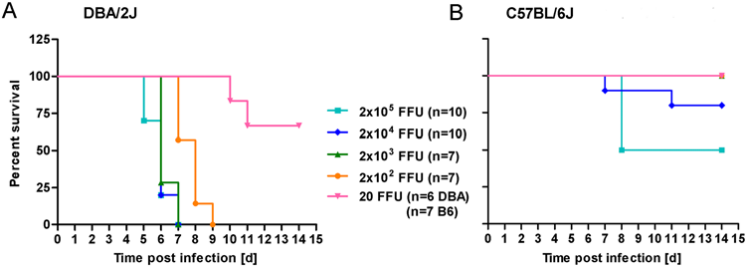
DBA/2J mice are highly susceptible to PR8 infections compared to C57BL/6J mice. DBA/2J (A) and C57BL/6J (B) mice were infected with increasing doses of PR8 virus via the intranasal route and survival was recorded for the following 14 days. Mortality includes also mice that were sacrificed because they had lost more than 25% of body weight.

Escalating doses of virus inoculates were applied to C57BL/6J and DBA/2J mice to determine the relative range of susceptibility. As shown in figure 3, DBA/2J mice died at very low viral doses (LD_50_ of 36 FFU) whereas all C57BL/6J mice survived doses of up to 10^3^ FFU. In this particular experiment, half of the C57BL/6J mice died at an FFU of 2×10^5^. However, as mice had to be sacrificed as part of the protocol when the weight loss was more than 25%, the threshold for the lethal dose varied in C57BL/6J mice. In other experiments with doses of 2×10^5^ FFU, C57BL/6J mice lost weight close to 25% but did not exceed this threshold and were recorded as survivors. Therefore, we conclude that the LD_50_ for C57BL/6J is above or equal to 2×10^5^ FFU. It thus exceeds the LD_50_ for DBA/2J mice by at least a factor of 10^3^.

**Figure 4:**
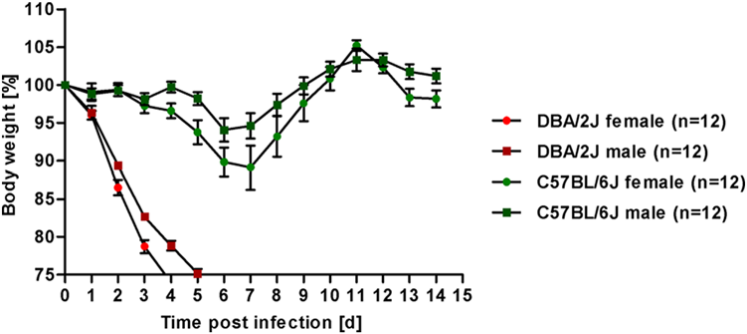
Male and female mice of DBA/2J and C57BL/6J mice show similar weight loss and survival after infection with Influenza A virus. DBA/2J female, DBA/2J male, C57BL/6J female and C57BL/J male were infected intra-nasally with 2×10^3^ FFU PR8 virus. Weight loss and survival of infected mice was followed over a period of 14 days. Mortality includes also mice that were sacrificed because they had lost more than 25% of body weight. Mean percent of body weight change (±SEM) is shown. p-values for significance were calculated for pair wise comparison between all groups and for all days using non-parametric Mann-Whitney-U-test. On days 2–4, all DBA/2J male and female groups differed significantly in their weight loss from the C57BL/6J groups (p<0.001 for all comparisons). No consistently significant difference was observed between male and female C57BL/6J groups (except at day 4, p<0.05), whereas the male and female DBA/2J groups were significantly different at days 2–4 (p<0.05 at day 2 and p<0.01 at days 3, 4).

For infectious diseases, differences between sexes are observed in several cases. We therefore compared the susceptibility to influenza infections in male and female mice. As illustrated in figure 4, male and female mice from the DBA/2J strain showed similar weight loss kinetics and were both highly susceptible. Both male and female mice from the C57BL/6J strain were resistant. Slight differences in the weight loss curves were observed between male and female mice in both strains, but they were not significant for the C57BL/6J groups. For DBA/2J mice significant differences were observed at days 2–4 after infection.

**Figure 5:**
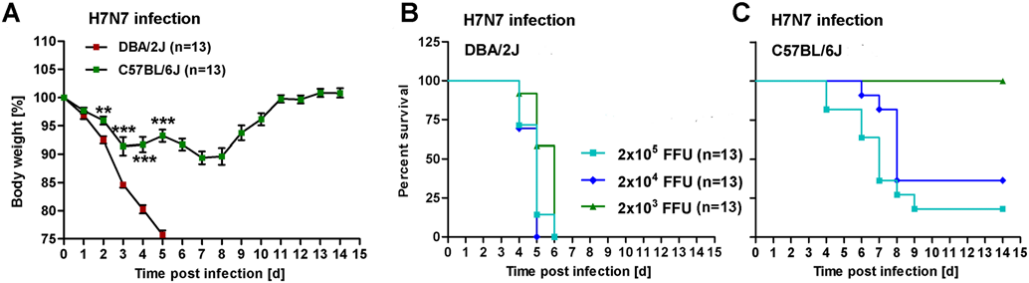
DBA/2J mice are highly susceptible to H7N7 (SC35M) virus infection compared to C57BL/6J mice. (A): DBA/2J and C57BL/6J mice were infected intra-nasally with 2×10^3^ FFU SC35M virus. DBA/2J (B) and C57BL/6J (C) mice were infected with increasing doses of SC35M (H7N7) virus via the intra-nasal route. Weight loss and survival of infected mice was followed over a period of 14 days. Mortality includes also mice that were sacrificed because they had lost more than 25% of body weight. Data are from two independent experiments. Mean percent of body weight change (±SEM) is shown. DBA/2J and C57BL/6J groups were compared for statistically significant differences using non-parametric Mann-Whitney-U-test. *: p value<0.05; **: p<0.01; ***: p value<0.001.

The PR8 (H1N1) influenza A virus was initially isolated from a human and subsequently adapted to mice [39]. To exclude that the observed mouse strain differences are specific for only one influenza subtype, we infected C57BL/6J and DBA/2J mice with another mouse-adapted virus subtype, SC35M. This virus represents an H7N7 influenza A virus subtype, was originally isolated from seal and then adapted to mouse [40]. After intra-nasal infection with SC35M virus, DBA/2J mice exhibited the same high susceptibility (figure 5A) as observed after infection with the PR8 virus, and C57BL/6J mice were much more resistant than DBA/2J mice. However, compared to infections with PR8 (figures 1 and 3), C57BL/6J mice started to die at lower doses of infection with SC35M and exhibited a slightly different weight loss kinetics (figure 5C). The peak weight loss was still observed between days 7 and 8. But in contrast to infections with PR8, infection of C57BL/6J mice with SC35M resulted in an early weight loss during the first three days, although at this infection dose, none of the C57BL/6J mice died. The C57BL/6J mice then transiently recovered and lost weight again with a kinetics similar to the weight loss observed for PR8 infections.

### Early differences in viral loads are present between C57BL/6J and DBA/2J mouse strains

**Figure 6:**
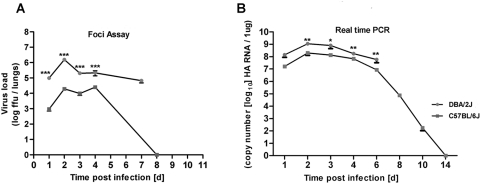
Higher viral load is detected in DBA/2J mice compared to C57BL/6J mice. DBA/2J and C57BL/6J mice were infected intra-nasally with 2×10^3^ FFU and viral load was determined at the indicated times post inoculation for infectious particles measured by foci assay (A) or by copy number of viral hemagglutinin (HA) RNA (B). Mean +/− SEM are shown. For foci assay in (A), 9 DBA/2J mice were used at all time points, and for C57BL/6J, 6 mice were used at day1, 8 mice at day 2 and 9 mice at days 3, 4, and 8. For RNA assays in (B), 10 mice were used except 15 for day 4, and 5 for day 6. DBA/2J and C57BL/6J mice were compared for statistical significant differences using non-parametric Mann-Whitney-U-test. *: p value<0.05; **: p<0.01; ***: p value<0.001.

The extreme susceptibility of DBA/2J mice could be due to enhanced viral replication and associated tissue damage or a detrimental immunopathology of the host response, or both. Therefore, we analyzed viral loads by determining infectious particles and viral mRNA copies of the hemagglutinin (HA) gene in the lung. On days 1 and 2, the amount of infectious virus particles was about 100- and 80-fold higher in DBA/2J mice compared to C57BL/6J mice (figure 6A). This difference decreased to about 20 and 10-fold on days 3 and 4, respectively (figure 6A). In the surviving C57BL/6J mice, virus titers were below the level of detection at day 8 (figure 6A). Similarly, viral HA mRNA was between 5- and 9-fold higher in DBA/2J mice compared to C57BL/6J mice at days 1–3 and at day 6 (figure 6B). At day 4 after infection, this difference was less pronounced (2.6-fold) (figure 6B). The reduced virus replication in DBA/2J mice on days 4 and 6 was most probably due to the severe pathology in the lungs of these mice (see also histological studies) resulting in epithelial cell necrosis which does not allow any further increase in virus replication. In C57BL/6J mice, copy numbers of viral HA RNA decreased considerably after day 6 and were below the level of detection on day 14 (figure 6B).

### Expression of inflammatory cytokines is higher in DBA/2J mice

**Figure 7:**
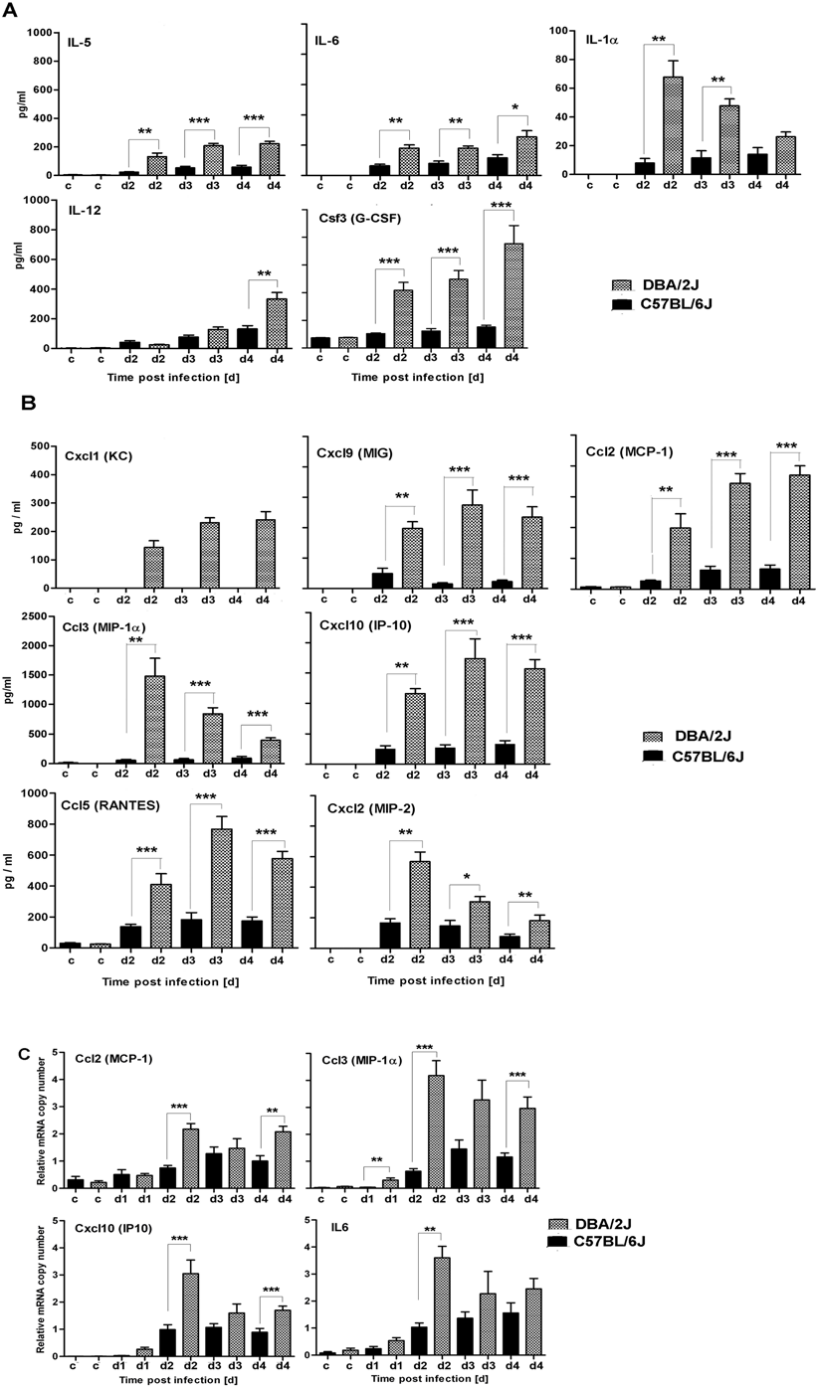
DBA/2J mice exhibit a stronger inflammatory response than C57BL/6J mice. DBA/2J (checked bars) and C57BL/6J mice (black bars) were infected intra-nasally with 2×10^3^ FFU of PR8 virus. Bronchio-alveolar lavage (BAL) was collected from non-infected controls (c) or at the indicated days (d1, d2, d3, d4) post infection, and the concentration of cytokines (A) or chemokines (B) was determined. Expression of cytokines and chemokines was determined by real-time PCR (C). Each time point represents the mean value ±SEM of 7 mice per group for (A) and (B), and of 10 mice per group for (C). DBA/2J and C57BL/6J mice were compared for statistically significant differences using non-parametric Mann-Whitney-U-test. *: p value<0.05; **: p<0.01; ***: p value<0.001.

During the course of an infection, the host responds with the production of various cytokines and chemokines which then activate the different components of the innate and adaptive immune system. Therefore, the presence of several cytokines and chemokines was studied in broncho-alveolar lavages (BAL) of infected C57BL/6J and DBA/2J mice. In total, 22 cytokines and chemokines were analyzed. As shown in figure 7A, the cytokines Il5, Il6, Il1α, Il12, and Csf3 (G-CSF) were elevated in the lungs of infected compared to non-infected mice. In all cases, levels of expression were higher in DBA/2J than in C57BL/6J mice. Similarly, the chemokines Ccl2 (MCP-1), Ccl3 (MIP-1α), Ccl5 (RANTES), Cxcl1 (KC), Cxcl2 (MIP-2), Cxcl9 (MIG), and Cxcl10 (IP-10) were higher in infected, compared to non-infected DBA/2J and C57BL/6J mice (figure 7B). DBA/2J mice exhibited a higher level of expression for all chemokines tested. Also, at the transcriptional level, elevated expression of chemokines and cytokines in DBA/2J compared to C57BL/6J mice was observed by real-time PCR analysis for *Ccl2*, *Ccl3*, *Cxcl10* and *Il6* (figure 7C).

### Severe damage of bronchial epithelia occurs in DBA/2J mice

**Figure 8:**
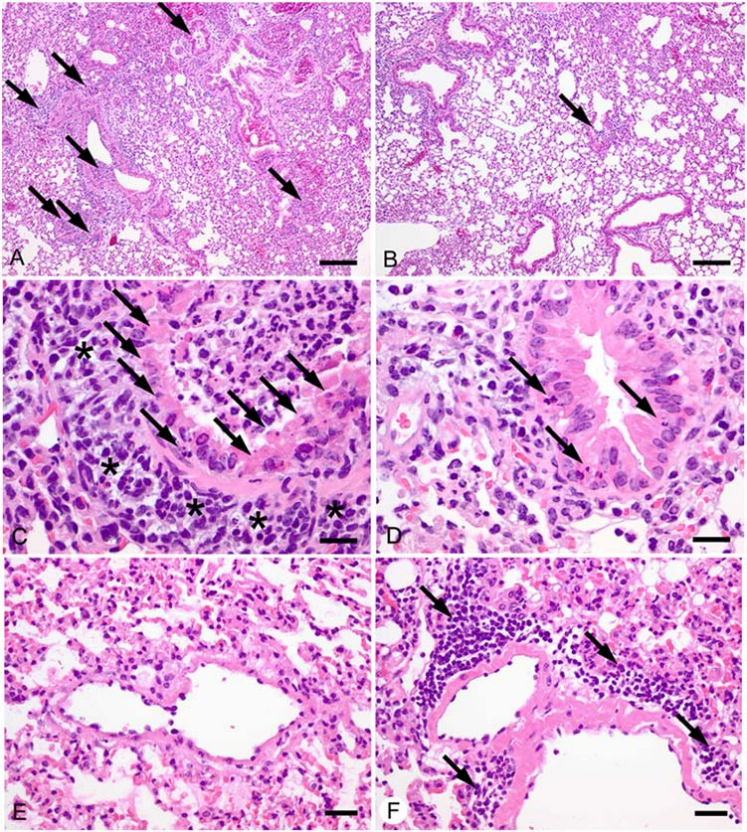
Severe damage of bronchial epithelia occurs in DBA/2J compared to C57BL/6J mice. DBA/2J (A, C, E) and C57BL/6J mice (B, D, F) were infected intra-nasally with 2×10^3^ FFU of PR8 virus. Lung sections were stained with hematoxylin and eosin. A, B: Two days after infection, the lungs of DBA/2J mice (A) were more consolidated with higher numbers of plugged airways (arrows) than C57BL/6J mice (B). C, D: The bronchioli and bronchi of DBA/2J mice (C) were plugged with degenerate bronchial epithelial cells and neutrophils with higher degrees of degeneration, necrosis and loss of epithelial cells (arrows) two days after infection. In addition, the airways were surrounded by larger numbers of neutrophils and macrophages (asterisks). Airways of C57BL/6J mice (D) showed less damage with little or no plugging of airways. At that time point, the lungs of both strains had few infiltrations with lymphocytes. E, F: Four days after infection, virtually no extravasations of lymphocytes were detected in DBA/2J mice (E) whereas marked perivascular lymphocytic infiltrations (arrows) were observed in the pulmonary interstitium of C57BL/6J mice (F). Bars  =  250 µm (A, B), 25 µm (C, D) and 50 µm (E, F).

Histological analyses of infected mice revealed striking differences between the tissue lesions in DBA/2J and C57BL/6J mice. The overall lung tissues were more densely consolidated with larger numbers of affected airways in DBA/2J mice compared to C57BL/6J mice (figure 8A, B). In both mouse strains, bronchial and bronchiolar epithelial cells showed degeneration, necrosis and loss with accumulation of sloughed cells and mostly degenerate neutrophils in the airway lumen at days 2 and 3 after infection. However, the degree of bronchial epithelial necrosis and airway plugging by cellular debris was much more pronounced in DBA/2J than C57BL/6J mice. This was accompanied by larger numbers of neutrophils and macrophages around the affected airways in DBA/2J mice (figure 8C, D). On the other hand, C57BL/6J mice developed a much stronger perivascular and peribronchial infiltration of lymphocytes at day 4 after infection (figure 8E, F). Alveolar epithelial cells were mostly unaffected. Few macrophages and virtually no plasma cells were seen in the lungs, with no differences between the two mouse strains at days 2 to 4 post infection.

## Discussion

Studies in mouse model systems have revealed that hundreds of genes are involved in the host defense against microbial infections and that the interaction of these genes and pathways is very complex [27], [31]–[37]. Although several single gene mutations are known which confer resistance or susceptibility to an infection with influenza A virus, very few studies have addressed the influence of multiple complex genetic interactions in mouse genetic reference populations [29], [41]. Therefore, as a first step towards the understanding of complex genetic traits involved in the host response to influenza infections, we have studied the susceptibility to infection with H1N1 and H7N7 influenza virus subtypes in different inbred laboratory mouse strains.

Our studies reveal that inbred mouse strains exhibit large differences in their host response to an infection with the H1N1 influenza A virus (PR8). Both the time course of the weight loss as well as survival rate was strikingly different between different laboratory mouse strains. These results demonstrate a strong genetic influence on the host susceptibility to influenza virus infections. We hypothesize that the different kinetics of the weight loss curves indicate that different mouse strains mount different qualitative, quantitative and temporal profiles of the host defense. For example, C57BL/6J mice were mostly affected at the point which correlates with the activity of TRAIL-expressing influenza-specific CD8 T cells infiltrating the infected lungs [42]. On the other hand, BALB/cByJ, CBA/J and SJL/JOrlCrl mouse strains showed weight loss early after infection, at a time when the peak of virus replication in the respiratory epithelium is observed.

Most notably, two mouse strains, DBA/2J and A/J, exhibit an extremely high susceptibility to influenza infections. All infected animals died within the first seven days after inoculation with low virus doses. The other strains tested showed 100% survival under these conditions. The LD_50_ for the highly susceptible mouse strain DBA/2J was more than three orders of magnitude lower compared to the resistant strain C57BL/6J. DBA/2J mice were highly susceptible to both the H1N1 (PR8) and H7N7 influenza virus (SC35M) subtypes. Thus, DBA/2J mice seem to exhibit a general high susceptibility to influenza virus infections, independent of the virus subtype.

To our knowledge, this is the first study that directly compares different inbred mouse laboratory strains for their susceptibility to influenza A virus. Most laboratories have used BALB/c mice or, in the context of knock-out mutants, C57BL/6J mice. So far, direct comparisons of the susceptibility of inbred strains were only performed for BALB/cByJ and C57BL/6J [29]. The analysis of gene expression patterns in this previous study revealed that more than 1000 genes were differentially expressed after infection with influenza virus A/HKX31 (H3N2).

DBA/2J mice were also used in comparison to other inbred mouse strains with regard to their susceptibility to infections with other pathogens. After infection with group A streptococci, DBA/2J belonged to the group of resistant mice [43], [44], whereas after infection with *Mycobacterium tuberculosis*, these mice were amongst the susceptible strains [45], [46]. Thus, DBA/2J mice do not appear to suffer from a general immune deficiency.

We performed a more detailed comparison between a susceptible strain, DBA/2J and a resistant strain, C57BL/6J, to gain further insight into the cellular and molecular factors that may contribute to the high susceptibility of DBA/2J mice to influenza infection. In infected DBA/2J mice, we observed higher virus replication at early time points after infection and a much stronger immune response than in C57BL/6J infected mice. Histological analyses showed that at day 4 after infection, the damage of the bronchial epithelium was being repaired in C57BL/6J mice but DBA/2J mice still showed the same severe lung phenotype as on day 2. Thus, the most likely explanation for the high susceptibility in DBA/2J mice is that both the continuous high level of viral replication and associated destruction of the lung epithelium as well as a highly activated and detrimental immune response lead to the lethal outcome of the infection. Several studies have demonstrated that both these factors can contribute to influenza induced lung pathology [5].

At present, we do not have an explanation for the rapid accumulation of virus at early time points after infection in DBA/2J mice. This effect may be due to a high replication rate in epithelial cells as it has been shown for a highly virulent PR8 variant [47]. Alternatively, receptors for virus entry into epithelial cells may be more densely distributed or exhibit a more favorable structure in DBA/2J mice. We have initiated studies to investigate in more detail the spatial distribution and individual cellular viral loads of infected cells in the lungs, as well as comparing the rate of replication in mouse embryonic fibroblasts.

The analysis of broncho-alveloar fluid and transcripts in the lung revealed that many cytokines and chemokines were expressed in both C57BL/6J and DBA/2J mice after infection. Thus, DBA/2J mice were able to mount a normal, early immune defense. However, susceptible DBA/2J mice exhibited a much stronger inflammatory response than resistant C57BL/6J mice. The CXC-chemokines Cxcl1, Cxcl2, Cxcl9 and Cxcl10 were found to be secreted into the broncho-alveolar space at a higher level in DBA/2J than in C57BL/6J mice. Cxcl9 and Cxcl10 chemokines target the Cxcr3 receptor expressed on activated T cells, whereas the Cxcl2 chemokine targets the Cxcr2 receptor expressed on neutrophils [48]. Of the CC chemokines, Ccl2, Ccl3 and Ccl5 were found to be elevated in DBA/2J mice. Both chemokines target receptors on macrophages, T cells, NK cells, granulocytes and dendritic cells [49]. Similarly, the cytokines Il1α, Il5, Il6, and Il12 were secreted into the broncho-alveolar space of both strains after infection but were higher in DBA/2J mice. The inflammatory protein Tnfα was found consistently, although at a low level in DBA/2J but not in C57BL/6J mice (data not shown). The production of cytokines and chemokines as a response to influenza A infection in mice and humans has been described in various studies (*e.g.* [5], [50], [51]) and it has been postulated that a strong innate immune response may cause severe detrimental immune pathologies [5]. Therefore, these observations indicate that the strong early inflammatory response contributes to the severe lung pathology and lethality in DBA/2J mice.

The strongly elevated inflammatory host response and the associated severe lung pathology observed in DBA/2J mice in this study are very similar to the response to infection of resistant BALB/c and C57BL/6J mice with highly pathogenic viruses. Strongly elevated levels of cytokines and chemokines have been found in infections with the highly pathogenic H5N1 avian influenza virus and with the 1918 virus of the Spanish flu compared to infections with less virulent virus subtypes [14], [15], [52]. In addition viral loads were higher in mice infected with highly pathogenic viruses [52], findings that correlate with those in human patients [5]. Based on these observations, it has been hypothesized that highly pathogenic avian viruses cause a “cytokine storm” which results in detrimental immune responses, although this has recently been questioned in connection with H5N1 virus [53].

Several single gene loci have been studied in mouse knock-out or natural mutants in the context of influenza infections. *Mx1* is mutated in most laboratory strains but fully functional in several wild-derived mice [54]–[57]. *Mx1* represents the major effector of the interferon response by inhibiting viral replication [54]. The mouse strains used in our study all carry a mutated *Mx1* allele [55], except for FVB for which the status has currently not been characterized. Since both DBA/2J and C57BL/6J mice are deficient for *Mx1*, yet DBA/2J is highly susceptible, we hypothesize that the high susceptibility of the DBA/2J mice to influenza virus may be due to differences in pathways that are not downstream of the interferon response.

Our findings now provide a basis for the mapping of additional genomic regions underlying host susceptibility to influenza infections. F1 hybrids from a cross between DBA/2J and C57BL/6J exhibited an intermediate weight loss phenotype and were resistant at a dose of 2×10^3^. We thus speculate that the susceptibility to influenza infection is a polygenic trait. We have initiated F2-backcrosses and an analysis of the BXD recombinant inbred strain set (generated from the parental strains DBA/2J and C57BL/6J; [58], [59]) to further narrow down the genomic regions responsible for the high susceptibility in DBA/2J mice.

In conclusion, both the continuously high viral load and the hyper-reactive inflammatory response appear to be the main causes for the high susceptibility and lethal outcome in DBA/2J mice after influenza A infections. DBA/2J mice exhibited an enhanced immune response which was similar to a host infected with a highly virulent 1918-H1N1 or H5N1 influenza virus [5], [17]. We thus expect that further studies aimed at unraveling the differential host responses in inbred mouse strains at the cellular, genetic and molecular level will not only allow identification of critical genomic regions of susceptibility but also contribute to a better understanding of the pathology associated with infections with high pathogenic influenza A virus subtypes.

## Materials and Methods

### Virus, mouse strains and infections

Mouse-adapted virus strains, influenza A/Puerto Rico/8/34 (H1N1; PR8) and influenza A/Seal/Massachussetts/1/80 (H7N7; SC35M), were propagated in the chorio-allantoic cavity of 10-day-old embryonated hen eggs for 48 hours at 37°C. Inbred mouse strains C57BL/6J, DBA/2J, FVB/NJ, CBA/J, BALB/cByJ, and (C57BL/6J×DBA/2J) F1 were obtained from Janvier, France, SJL/JOrlCrl from Charles River, Germany; DBA/2NHsd from Harlan, Germany, and A/JOlaHsD (A/J) from Harlan, U.K. Mice were maintained under specific pathogen free conditions and according to the German animal welfare law. All experiments were approved by an external committee according to the German regulations on animal welfare. For infection experiments, mice were anesthetized by intra-peritoneal injection with Ketamin-Rompun with doses adjusted to the individual body weight. Virus was administered intra-nasally in a total volume of 20 µl sterile PBS. Weight loss and survival of infected mice was followed over a period of 14 days. In addition to mice that were found dead, mice with a weight loss of more than 25% of the starting bodyweight were euthanized and recorded as dead.

### Extraction of RNA and real-time PCR

Total RNA was prepared from lungs using the RNeasy Midi kit (Qiagen, Hilden, Germany) following the manufacturer's protocol. Quality and quantity check was performed by using Agilent 2100 bioanalyzer. For determination of viral load, reverse transcription was carried out according to the manufacturer's instructions using the ThermoScript™ RT-PCR kit (Invitrogen, Carlsbad, USA). Briefly, 500 ng lung-extracted RNA and random hexamer primers (Invitrogen) were mixed and denatured at 70°C for 8 min, followed by reverse transcription at 60°C for 1 h. Reactions were terminated by incubating the mixture at 85°C for 5 min and RNase H treatment at 37°C for 20 min. Samples were diluted to a final volume 50 µl and stored at −20°C. 5 µl of cDNA product were amplified with specific primers. For HA analysis, the following primers were used: HA01 (5′ CCAGAATATACACCCAGTCACAAT 3′) and HA02 (5′ GATCCGCTGCATAGCCTGAT 3′). For the external standard curve, serial dilutions (between 10^10^ and 10^2^ molecules) of *in vitro* transcribed pGEM-T Easy-HA RNA were used. For determination of cytokine expression, RNA was reversely transcribed with a SuperScriptII reverse transcriptase kit (Invitrogen) according to the manufacturer's protocol. 500 ng lung-extracted RNAs and random hexamers (Invitrogen) were mixed and denatured at 70°C for 10 minutes, followed by reverse transcription at 42° for 1 h. For cytokines and chemokines analyses the following primers were used: *Rps9* (5′ CTGGACGAGGGCAAGATGAAGC, 3′ TGACGTTGGCGGATGAGCACA), *Il-6* (5′ TAACAAGAAAGACAAAGCCAGAGT, 3′ TTGGAAATTGGGGTAGGAAAG), *Cxcl10* (5′ CTCTCCATCACTCCCCTTTACCC, 3′ GCTTCGGCAGTTACTTTTGTCTCA), *Il-1 β* (5′ ACTACAGGCTCCGAGATGAACAAC, 3′ CCCAAGGCCACAGGTATTTT), *Mip-1α* (5′ CTCCCAGCCAGGTGTCATTTTC, 3′ CTCAGGCATTCAGTTCCAGGTCAG), *Ccl2* (5′ CATGCTTCTGGGCCTGCTGTT, 3′ CCTGCTGCTGGTGATCCTCTTGTA). Real-time PCR was carried out with the DNA Master SYBR Green I kit (Roche, Mannheim, Germany) using a LightCycler 480 apparatus (Multiwell Plate 96, Roche). The housekeeping ribosomal protein S9 (*Rps9*) gene was used for normalization.

### Virus titration by foci assay

MDCK II cells (American Type Culture Collection, Manassas, USA) were cultured at 37°C in 5% CO_2_ in Dulbecco's Modified Eagle Medium (DMEM), supplemented with 10% fetal calf serum (FCS), 1% penicillin/streptomycin. 1×10^5^ cells were seeded in 96-well culture plates and incubated at 37°C in 5% CO_2_ for 24 h. For foci assay, lungs of mice were homogenized in phosphate buffered saline (PBS) with 0.1% BSA using the PolyTron 2100 homogenizer. Debris was removed by centrifugation for 10 min at 1000 rpm. The samples were aliquoted and stored at −70°C. Serial 10-fold dilutions of lung homogenates in DMEM containing 0.1% BSA were prepared and added to MDCK II cells. After 1 h, cells were washed twice with PBS and fixed with 4% formalin in PBS (100 µl/well). The plates were incubated at 37°C in 5% CO_2_ for 1 h. The inoculates were aspirated and replaced with 100 µl of 1% Avicell overlay and incubated at 37°C for 24 h. Subsequently, the plates were washed twice with PBS and fixed with 4% formalin in PBS for 10 min at room temperature. The formalin was removed and the cells were washed and incubated for 10 min with 100 µl/well Quencher (0.5% Triton ×100, 20 mM glycine in PBS). After 10 min the cells were washed with Wash Buffer (0,5% Tween 20 in PBS) and blocked with 50 µl Blocking Buffer (0.5% Tween 20, 1% BSA in PBS) at 37°C in 5% CO_2_ for 30 min. The primary antibody (anti-influenza Nucleocapsid NP polyclonal goat antibody from Virostat, Portland, USA) and the secondary antibody (anti-goat-HRP from KPL, Gaithersburg MD, USA) were diluted 1∶1000 in Blocking Buffer. 50 µl of the primary antibody were added to each well and incubated at room temperature for 1 h. After 1 h, the cells were washed three times with Wash Buffer, incubated with 50 µl of the secondary antibody for 45 min, washed again and incubated with 50 µl of substrate (True Blue from KPL) until the blue spots from infected cell foci appeared. Foci were counted and viral titers were calculated as focus forming units (FFU/lung).

### Cytokine and chemokine analysis

Mice were euthanized with CO_2_. A sterile, 22-gauge catheter was inserted into the exposed tracheal lumen. Broncho-alveolar lavage fluid was collected from two 0.5 ml instillations of PBS containing 2 mM EDTA per mouse. Supernatants were stored at −20°C. Cytokine and chemokine levels were analyzed using the multi-plex cytokine analysis kit from Biosource (Carlsbad, USA) following the manufacturer's instructions. The plates were read on a Luminex 100™ instrument. MIP-2 and IL-10 (Biorad, Hercules, CA, USA) and G-CSF and RANTES (Invitrogen) were analyzed separately in quadruplet following the manufacturer's instructions.

### Histological analyses

Mice were euthanized with CO_2_. Lungs were prepared and immersion-fixed for 24 h in 4% buffered formaldehyde solution (pH 7.4), dehydrated in a series of graded alcohols and embedded in paraffin. Sections (3 µm) were cut from three evenly distributed levels of the paraffin blocks and stained with hematoxylin and eosin. Histological sections were examined and graded by two pathologists in a blind fashion.

### Statistical test

Means±SEM were calculated and the data for percent body weights, viral titers, and cytokines/chemokines data were evaluated for statistical significant differences by the non-parametric Mann-Whitney-U-test using GraphPad Prism version 5.00 for Windows (GraphPad Software, San Diego, California; www.graphpad.com). p values of ≤0.05 were considered significant.

## References

[1] Johnson NP, Mueller J (2002). Updating the accounts: global mortality of the 1918–1920 “Spanish” influenza pandemic.. Bull Hist Med.

[2] Kilbourne ED (2006). Influenza pandemics of the 20th century.. Emerg Infect Dis.

[3] Rajagopal S, Treanor J (2007). Pandemic (avian) influenza.. Semin Respir Crit Care Med.

[4] Fauci AS (2006). Seasonal and pandemic influenza preparedness: science and countermeasures.. J Infect Dis.

[5] La Gruta NL, Kedzierska K, Stambas J, Doherty PC (2007). A question of self-preservation: immunopathology in influenza virus infection.. Immunol Cell Biol.

[6] Taubenberger JK, Morens DM (2008). The pathology of influenza virus infections.. Annu Rev Pathol.

[7] Obenauer JC, Denson J, Mehta PK, Su X, Mukatira S (2006). Large-scale sequence analysis of avian influenza isolates.. Science.

[8] Webster RG, Bean WJ, Gorman OT, Chambers TM, Kawaoka Y (1992). Evolution and ecology of influenza A viruses.. Microbiol Rev.

[9] Casanova JL, Abel L (2004). The human model: a genetic dissection of immunity to infection in natural conditions.. Nat Rev Immunol.

[10] Hill AV (2006). Aspects of genetic susceptibility to human infectious diseases.. Annu Rev Genet.

[11] Sorensen TI, Nielsen GG, Andersen PK, Teasdale TW (1988). Genetic and environmental influences on premature death in adult adoptees.. N Engl J Med.

[12] Albright FS, Orlando P, Pavia AT, Jackson GG, Cannon Albright LA (2008). Evidence for a heritable predisposition to death due to influenza.. J Infect Dis.

[13] Gottfredsson M, Halldorsson BV, Jonsson S, Kristjansson M, Kristjansson K (2008). Lessons from the past: familial aggregation analysis of fatal pandemic influenza (Spanish flu) in Iceland in 1918.. Proc Natl Acad Sci U S A.

[14] Kash JC, Tumpey TM, Proll SC, Carter V, Perwitasari O (2006). Genomic Analysis of Increased Host Immune and Cell Death Responses Induced by 1918 Influenza Virus.. Nature.

[15] Lipatov AS, Andreansky S, Webby RJ, Hulse DJ, Rehg JE (2005). Pathogenesis of Hong Kong H5N1 Influenza Virus Ns Gene Reassortants in Mice: the Role of Cytokines and B- and T-Cell Responses.. Journal of General Virology.

[16] Tate MD, Brooks AG, Reading PC (2008). The role of neutrophils in the upper and lower respiratory tract during influenza virus infection of mice.. Respir Res.

[17] Tumpey TM, Garcia-Sastre A, Taubenberger JK, Palese P, Swayne DE (2005). Pathogenicity of Influenza Viruses With Genes From the 1918 Pandemic Virus: Functional Roles of Alveolar Macrophages and Neutrophils in Limiting Virus Replication and Mortality in Mice.. Journal of Virology.

[18] Snelgrove RJ, Goulding J, Didierlaurent AM, Lyonga D, Vekaria S (2008). A critical function for CD200 in lung immune homeostasis and the severity of influenza infection.. Nat Immunol.

[19] GeurtsvanKessel CH, Willart MA, van Rijt LS, Muskens F, Kool M (2008). Clearance of influenza virus from the lung depends on migratory langerin+CD11b- but not plasmacytoid dendritic cells.. J Exp Med.

[20] McGill J, Van Rooijen N, Legge KL (2008). Protective influenza-specific CD8 T cell responses require interactions with dendritic cells in the lungs.. J Exp Med.

[21] Gazit R, Gruda R, Elboim M, Arnon TI, Katz G (2006). Lethal influenza infection in the absence of the natural killer cell receptor gene Ncr1.. Nat Immunol.

[22] Coro ES, Chang WL, Baumgarth N (2006). Type I IFN receptor signals directly stimulate local B cells early following influenza virus infection.. J Immunol.

[23] Stambas J, Guillonneau C, Kedzierska K, Mintern JD, Doherty PC (2008). Killer T cells in influenza.. Pharmacol Ther.

[24] Bergmann M, Garcia-Sastre A, Carnero E, Pehamberger H, Wolff K (2000). Influenza virus NS1 protein counteracts PKR-mediated inhibition of replication.. J Virol.

[25] Haller O, Arnheiter H, Lindenmann J (1976). Genetically determined resistance to infection by hepatotropic influenza A virus in mice: effect of immunosuppression.. Infect Immun.

[26] Koerner I, Kochs G, Kalinke U, Weiss S, Staeheli P (2007). Protective role of beta interferon in host defense against influenza A virus.. J Virol.

[27] Crozat K, Georgel P, Rutschmann S, Mann N, Du X (2006). Analysis of the MCMV resistome by ENU mutagenesis.. Mamm Genome.

[28] Marquis JF, Nantel A, LaCourse R, Ryan L, North RJ (2008). Fibrotic response as a distinguishing feature of resistance and susceptibility to pulmonary infection with Mycobacterium tuberculosis in mice.. Infect Immun.

[29] Ding M, Lu L, Toth LA (2008). Gene expression in lung and basal forebrain during influenza infection in mice.. Genes Brain Behav.

[30] Peters LL, Robledo RF, Bult CJ, Churchill GA, Paigen BJ (2007). The mouse as a model for human biology: a resource guide for complex trait analysis.. Nat Rev Genet.

[31] Beutler B, Eidenschenk C, Crozat K, Imler JL, Takeuchi O (2007). Genetic analysis of resistance to viral infection.. Nat Rev Immunol.

[32] Buer J, Balling R (2003). Mice, microbes and models of infection.. Nat Rev Genet.

[33] Campino S, Kwiatkowski D, Dessein A (2006). Mendelian and complex genetics of susceptibility and resistance to parasitic infections.. Semin Immunol.

[34] Fortin A, Abel L, Casanova JL, Gros P (2007). Host genetics of mycobacterial diseases in mice and men: forward genetic studies of BCG-osis and tuberculosis.. Annu Rev Genomics Hum Genet.

[35] Lengeling A, Pfeffer K, Balling R (2001). The battle of two genomes: genetics of bacterial host/pathogen interactions in mice.. Mamm Genome.

[36] Marquis JF, Gros P (2008). Genetic analysis of resistance to infections in mice: A/J meets C57BL/6J.. Curr Top Microbiol Immunol.

[37] Vidal SM, Malo D, Marquis JF, Gros P (2008). Forward genetic dissection of immunity to infection in the mouse.. Annu Rev Immunol.

[38] Vance RE, Jamieson AM, Cado D, Raulet DH (2002). Implications of CD94 deficiency and monoallelic NKG2A expression for natural killer cell development and repertoire formation.. Proc Natl Acad Sci U S A.

[39] Hoffmann E, Krauss S, Perez D, Webby R, Webster RG (2002). Eight-plasmid system for rapid generation of influenza virus vaccines.. Vaccine.

[40] Gabriel G, Dauber B, Wolff T, Planz O, Klenk HD (2005). The viral polymerase mediates adaptation of an avian influenza virus to a mammalian host.. Proc Natl Acad Sci U S A.

[41] Trammell RA, Toth LA (2008). Genetic susceptibility and resistance to influenza infection and disease in humans and mice.. Expert Rev Mol Diagn.

[42] Brincks EL, Katewa A, Kucaba TA, Griffith TS, Legge KL (2008). CD8 T cells utilize TRAIL to control influenza virus infection.. J Immunol.

[43] Hollingsworth JW, Whitehead G, Berman KG, Tekippe EM, Gilmour MI (2007). Genetic basis of murine antibacterial defense to streptococcal lung infection.. Immunogenetics.

[44] Medina E, Goldmann O, Rohde M, Lengeling A, Chhatwal GS (2001). Genetic control of susceptibility to group A streptococcal infection in mice.. J Infect Dis.

[45] Mitsos LM, Cardon LR, Fortin A, Ryan L, LaCourse R (2000). Genetic control of susceptibility to infection with Mycobacterium tuberculosis in mice.. Genes Immun.

[46] Mitsos LM, Cardon LR, Ryan L, LaCourse R, North RJ (2003). Susceptibility to tuberculosis: a locus on mouse chromosome 19 (Trl-4) regulates Mycobacterium tuberculosis replication in the lungs.. Proc Natl Acad Sci U S A.

[47] Grimm D, Staeheli P, Hufbauer M, Koerner I, Martinez-Sobrido L (2007). Replication Fitness Determines High Virulence of Influenza a Virus in Mice Carrying Functional Mx1 Resistance Gene.. Proceedings of the National Academy of Sciences of the United States of America.

[48] Murphy PM, Baggiolini M, Charo IF, Hebert CA, Horuk R (2000). International union of pharmacology. XXII. Nomenclature for chemokine receptors.. Pharmacol Rev.

[49] Janeway CA, T P, Walport M, Sholchik MJ (2001). Immunobiology.

[50] Wareing MD, Lyon AB, Lu B, Gerard C, Sarawar SR (2004). Chemokine expression during the development and resolution of a pulmonary leukocyte response to influenza A virus infection in mice.. J Leukoc Biol.

[51] Fadel Sa Fau - Bromley SK, Bromley Sk Fau - Medoff BD, Medoff Bd Fau - Luster AD, Luster AD (2008). CXCR3-deficiency protects influenza-infected CCR5-deficient mice from mortality.. Eur J Immunol.

[52] Perrone LA, Plowden JK, Garcia-Sastre A, Katz JM, Tumpey TM (2008). H5N1 and 1918 pandemic influenza virus infection results in early and excessive infiltration of macrophages and neutrophils in the lungs of mice.. PLoS Pathog.

[53] Droebner K, Reiling SJ, Planz O (2008). Role of hypercytokinemia in NF-{kappa}B p50 deficient mice after H5N1 influenza A virus infection.. J Virol.

[54] Haller O, Staeheli P, Kochs G (2007). Interferon-induced Mx proteins in antiviral host defense.. Biochimie.

[55] Staeheli P, Grob R, Meier E, Sutcliffe JG, Haller O (1988). Influenza virus-susceptible mice carry Mx genes with a large deletion or a nonsense mutation.. Mol Cell Biol.

[56] Staeheli P, Haller O, Boll W, Lindenmann J, Weissmann C (1986). Mx protein: constitutive expression in 3T3 cells transformed with cloned Mx cDNA confers selective resistance to influenza virus.. Cell.

[57] Vanlaere I, Vanderrijst A, Guenet JL, De Filette M, Libert C (2008). Mx1 causes resistance against influenza A viruses in the Mus spretus-derived inbred mouse strain SPRET/Ei.. Cytokine.

[58] Peirce JL, Lu L, Gu J, Silver LM, Williams RW (2004). A new set of BXD recombinant inbred lines from advanced intercross populations in mice.. BMC Genet.

[59] Taylor BA, Wnek C, Kotlus BS, Roemer N, MacTaggart T, Phillips SJ (1999). Genotyping new BXD recombinant inbred mouse strains and comparison of BXD and consensus maps.. Mamm Genome.

